# Freezing of Apheresis Platelet Concentrates in 6% Dimethyl Sulfoxide: The First Preliminary Study in Turkey

**DOI:** 10.4274/tjh.2014.0181

**Published:** 2016-02-17

**Authors:** Soner Yılmaz, Rıza Aytaç Çetinkaya, İbrahim Eker, Aytekin Ünlü, Metin Uyanık, Serkan Tapan, Ahmet Pekoğlu, Aysel Pekel, Birgül Erkmen, Uğur Muşabak, Sebahattin Yılmaz, İsmail Yaşar Avcı, Ferit Avcu, Emin Kürekçi, Can Polat Eyigün

**Affiliations:** 1 Gülhane Military Medical Academy, Blood Training Center and Blood Bank, Ankara, Turkey; 2 Gülhane Military Medical Academy, Division of Pediatric Hematology, Ankara, Turkey; 3 Gülhane Military Medical Academy, Department of General Surgery, Ankara, Turkey; 4 Gülhane Military Medical Academy, Department of Medical Biochemistry, Ankara, Turkey; 5 Gülhane Military Medical Academy, Division of Immunology and Allergy, Ankara, Turkey; 6 Gülhane Military Medical Academy, Division of Hematology, Ankara, Turkey; 7 Gülhane Military Medical Academy, Department of Infectious Disease and Clinical Microbiology, Ankara, Turkey

**Keywords:** Frozen platelets, Flow-cytometric analysis, In vivo thrombin generation test

## Abstract

**Objective:**

Transfusion of platelet suspensions is an essential part of patient care for certain clinical indications. In this pioneering study in Turkey, we aimed to assess the in vitro hemostatic functions of platelets after cryopreservation.

**Materials and Methods:**

Seven units of platelet concentrates were obtained by apheresis. Each apheresis platelet concentrate (APC) was divided into 2 equal volumes and frozen with 6% dimethyl sulfoxide (DMSO). The 14 frozen units of APCs were kept at -80 °C for 1 day. APCs were thawed at 37 °C and diluted either with autologous plasma or 0.9% NaCl. The volume and residual numbers of leukocytes and platelets were tested in both before-freezing and post-thawing periods. Aggregation and thrombin generation tests were used to analyze the in vitro hemostatic functions of platelets. Flow-cytometric analysis was used to assess the presence of frozen treated platelets and their viability.

**Results:**

The residual number of leukocytes in both dilution groups was <1x106. The mean platelet recovery rate in the plasma-diluted group (88.1±9.5%) was higher than that in the 0.9% NaCl-diluted group (63±10%). These results were compatible with the European Directorate for the Quality of Medicines quality criteria. Expectedly, there was no aggregation response to platelet aggregation test. The mean thrombin generation potential of post-thaw APCs was higher in the plasma-diluted group (2411 nmol/L per minute) when compared to both the 0.9% NaCl-diluted group (1913 nmol/L per minute) and the before-freezing period (1681 nmol/L per minute). The flow-cytometric analysis results for the viability of APCs after cryopreservation were 94.9% and 96.6% in the plasma and 0.9% NaCl groups, respectively.

**Conclusion:**

Cryopreservation of platelets with 6% DMSO and storage at -80 °C increases their shelf life from 7 days to 2 years. Besides the increase in hemostatic functions of platelets, the cryopreservation process also does not affect their viability rates.

## Abstract (tr)

**Amaç:**

Trombosit süspansiyonlarının transfüzyonu, belirli klinik endikasyonlarda hastaların tedavisinin önemli bir parçasıdır. Bu çalışma ile Türkiye’de ilk kez olmak üzere trombositlerin in vitro hemostatik fonksiyonlarının kriyopreservasyon işleminden sonra değerlendirilmesi amaçlanmıştır.

**Gereç ve Yöntemler:**

Çalışmamızda 7 ünite trombosit süspansiyonu aferez yöntemiyle elde edildi. Her aferez trombosit konsantresi (ATK) iki eşit hacime ayrıldıktan sonra %6 dimetil sülfoksit (DMSO) kullanılarak donduruldu. Dondurulmuş 14 ünite ATK -80 °C’de bir gün süre ile bekletildi. ATK’lar 37 °C’de çözdürüldükten sonra otolog plazma veya %0,9 NaCl kullanılarak dilüe edildi. ATK’ların dondurma öncesi ve çözülme işlemi sonrası; hacim, rezidüel lökosit ve trombosit sayıları incelendi. Trombositlerin in vitro hemostatik fonksiyonların incelenmesinde agregasyon ve trombin jenerasyon testleri kullanıldı. Dondurma işlemine maruz kalan ATK’lardaki trombositlerin varlığı ve bu hücrelerin canlılığını değerlendirmek için akım sitometri yöntemi kullanıldı.

**Bulgular:**

Her iki dilüsyon grubunda yer alan ATK’ların residüel lökosit sayısı 1x106’nın altındaydı. Plazma ile dilüe edilen grubun ortalama trombosit geri kazanım oranı %0,9 NaCl ile dilüe edilen gruptan daha yüksekti (%88,1±9,5’e karşılık %63±10). Bu sonuçlar Avrupa İlaç Kalite ve Sağlık Hizmetleri Direktörlüğü’nün kalite kriterlerine uygundu. Trombosit agregasyon testine beklenildiği üzere yanıt alınamadı. Dondurulup çözülerek otolog plazma ile dilüe edilen ATK’ların ortalama trombin oluşturma potansiyeli (2411 nmol/L×dakika), %0,9 NaCl ile dilüe edilenlere (1913 nmol/L×dakika) ve dondurma işlemi öncesine göre (1681 nmol/L×dakika) daha yüksek saptandı. Kriyopreservasyon işlemi sonrası plazma ve %0,9 NaCl ile dilüe edilen ATK’ların akım sitometri yöntemi ile canlılığı sırasıyla %94,9 ve %96,6 olarak bulundu.

**Sonuç:**

Trombositlerin DMSO ile kriyopreserve edilerek -80 °C saklanmaları, raf ömürlerini 7 günden 2 yıla çıkarmaktadır. Kriyopreservasyon işlemi trombositlerin in vitro hemostatik fonksiyonlarını arttırmanın yanında canlılık oranlarını da etkilememektedir.

## INTRODUCTION

Currently in blood banking applications, platelet concentrates (PCs) prepared through apheresis or from buffy coat should be used within 5-7 days after preparation. In order to overcome the short shelf life-related problems, studies on frozen PCs have continued since the 1970s [1]. Although the literature data on the use of cryopreserved platelets showed that in vivo cryopreserved platelet suspensions have hemostatic activities superior to those of fresh apheresis suspensions, they showed delayed responses to in vitro platelet aggregation tests. Initially, this delay was attributed to the loss of aggregation capability of platelets during the cryopreservation process. However, recent studies proved that the in vitro failure of aggregation response to agonists occurred in response to the transformation of platelets into a procoagulant phenotype by the activation-degranulation process [2,3,4].

In the last decade, the demand for frozen platelets that have a long shelf life has increased for the treatment of military casualties in the Iraq and Afghanistan campaigns. As for Turkey, freezing and storing PCs as a part of contingency plans and prevention of this valuable blood product’s disposal due to short shelf life has an importance beyond emphasis.

The most commonly used cryopreservation protocol is the addition of dimethyl sulfoxide (DMSO) to PCs at a final concentration of 4%-6%, followed by removal of DMSO involving supernatant before the freezing process and finally freezing of the hyperconcentrated low volume of PCs. After the thawing process, PCs can be diluted by adding 0.9% NaCl, autologous plasma, or platelet additive solutions. This protocol can attain a gain of platelets between 70% and 80% [5]. In this study, we aimed to assess the in vitro hemostatic activity of cryopreserved platelets using different dilution methods (0.9% NaCl and autologous plasma).

## MATERIALS AND METHODS

In August 2013, Gülhane Military Medical Academy Ethics Committee Approval was received for the assessment of in vitro hemostatic activity of cryopreserved apheresis platelet concentrates (APCs). Written informed consent was obtained from all participants.

PCs were obtained using the apheresis method (Trima, Caridian BCT, Inc., Lakewood, CO, USA) from donors that met the National Blood and Blood Products criteria for the donation of APCs. APCs at a total of 200 mL were collected in acid-citrate-dextrose (ACD, NIH, Formula A, Baxter Healthcare Corp., Deerfield, IL, USA) at a ratio of 1 volume of ACD to 10 volumes of blood. APCs from all 7 donors were divided into 2 packs of 100 mL in volume each. One of each of the 100-mL packs was included in either the plasma-diluted group or the 0.9% NaCl-diluted group. Each group comprised 7 APCs. Before the freezing process, APCs were preserved in an automatic shaker on a horizontal plane at 20-24 °C for 1 day. Each of the APCs’ volume and weight were calculated at all stages of the procedure.

### Apheresis Platelet Concentrate Freezing Process

A 41-mL sample of plasma collected by apheresis from each donor and 0.9% NaCl were mixed with 9 mL of 27% DMSO in an empty blood bag located on a rigid ice pack for the plasma-diluted group and 0.9% NaCl-diluted groups, respectively. The resultant 50-mL mixture and 100 mL of APC were collected in a 750-mL ethyl vinyl acetate freezing bag (CryoMACS® Freezing Bag 750, Miltenyi Biotec, Teterow,Germany) through a sterile hose combining device. The final DMSO concentration in the freezing bag was 6% and the bag was centrifuged at 22 °C and 1250x g for 10 min (Thermo Fisher Scientific RC12BP, Asheville, NC, USA). A platelet pellet of 20-25 mL was obtained after removal of the supernatant and the bag was put into a cardboard freezing box and stored at -80 °C.

### Thawing of Frozen Apheresis Platelet Concentrates

The 1-day-old frozen APCs were thawed through immersion in 37 °C water within 10 min. Either 20 mL of autologous plasma or 0.9% NaCl was added to the APCs depending on the dilution group and they were kept at room temperature for 30 min.

### In Vitro Measurements

All analyses were repeated in the fresh state and after diluting the APCs in the post-thaw state.

### Residual Leukocyte and Platelet Counts

The frozen APCs were analyzed for the determination of platelet and residual leukocyte counts with a whole-blood analyzer device (ABX Pentra XL80, HORIBA ABX SAS, Montpellier, France).

### Platelet Aggregation Test

Platelet aggregation tests were performed with a Chrono-log platelet aggregometer by the same laboratory technician and thrombocyte agonist (ADP, epinephrine, collagen, and ristocetin) responses were assessed for both dilution groups.

### Thrombin Generation Test

Thrombin generation test (TGT) was performed with a calibrated automated thrombogram device (Thrombinoscope BV, Maastricht, the Netherlands) [6]. In this test, thrombin generation occurs in the co-presence of phospholipid and tissue factor present in the platelet supernatant and/or added reagents. The platelet-rich plasma reagent (Thrombinoscope BV) used in our test involves 1 pmol/L tissue factor. However, this reagent does not involve phospholipid and is used for assessing the presence of phospholipid in the medium. A sample of 80 µL was collected from both dilution groups. Each sample was transferred to 3 different microtitrated plates (Immulon 2 HB, Thermo Electron Corporation, Milford, MA, USA) that involved 20 µL of platelet-rich plasma reactant and 20 µL of thrombin calibrator. After the incubation of the mixture at 37 °C for 15 min, a 20-µL sample was collected and added to 20 µL of Fluo-buffer solution, and the reaction was monitored with a fluorometer. Using the Thrombinoscope program, the thrombogram curve, endogenous thrombin potential, and peak height were measured. The endogenous thrombin potential, which indicates the total amount of endogenous thrombin generated, was recorded as nmol/L per minute. The peak height, which indicates the highest measured value of thrombin, was shown as nmol/L.

### Flow Cytometry Analysis

Platelet samples were transferred to tubes containing K3 EDTA. CD41a FITC (BD Biosciences, San Jose, CA, USA) and 7-aminoactinomycin D (7-AAD) were used to determine viable platelets. The incubated cells were analyzed using the FACSDiva software for FACSCanto II model flow cytometry (BD Biosciences).

### Statistical Analysis

Quantitative results were presented as mean ± standard deviation and minimum-maximum. Categorical results were presented as number and percentage. All statistical analyses were processed using SPSS 14.0 for Windows (SPSS Inc., Chicago, IL, USA).

## RESULTS

The mean volume of APCs after dilution with autologous plasma or 0.9% NaCl was 45±3 mL. The mean platelet counts of the plasma and 0.9% NaCl groups were (123.6±13.7)x1011 (range: (106.9-143.5)x1011) and (84.6±7.6)x1011 (range: (77.8-100.8)x1011), respectively (Table 1). The freeze-thaw percentage recovery was calculated according to the standard operating procedure of the Naval Blood Research Laboratory [7]. The residual leukocyte counts of all APCs were <1x106. In the 0.9% NaCl-diluted group, one sample was excluded from the study due to damage to the plastic bag. The platelet and residual leukocyte counts and the rate of platelet recovery of APCs are shown in Table 1.

In all 13 fresh APC samples, platelet aggregation tests with ADP, epinephrine, and collagen were normal. There was no aggregation response to a variety of dilution ratios in any of the frozen treated platelet samples.

TGT revealed that post-thaw APCs diluted with autologous plasma (Figure 1A) had higher endogenous thrombin potentials when compared to fresh-state samples (Figure 1B) and post-thaw APC samples diluted with 0.9% NaCl (Figure 1C) (2411 vs. 1681 and 1913 nmol/L per minute). The peak height values were also higher in post-thaw APC samples diluted with autologous plasma (609 vs. 350 and 338 nmol/L, respectively).

As a result of flow-cytometric analysis, 99.2% of fresh APCs were stained with the CD41A thrombocyte indicator, while 97.9% were determined as viable when tested with 7-AAD nucleic acid dye. The viability rates of the post-thaw APC samples diluted with plasma and 0.9% NaCl are shown in Table 2.

## DISCUSSION

In 1956, Klein et al. reported the use of previously frozen platelets in an actively bleeding thrombocytopenic patient, and since then numerous studies have been reported on both the in vitro and in vivo efficacies of cryopreserved platelets [8]. Since Schiffer et al.’s 1976 study on the use of autologous platelets for the treatment of patients with leukemia, relevant studies until the 1990s showed that the platelets were damaged to a significant extent by the freezing process, which decreased their efficacy when compared to fresh platelets [1]. These results were supported by other in vitro studies that assessed the platelets’ primary hemostatic functions [1,9,10]. However, Khuri et al.’s 1999 report caused a shift in this paradigm; they showed that the in vivo hemostatic functions of cryopreserved APCs were superior to those of fresh preserved platelets [3]. Almost simultaneously, Bernard et al. reported the procoagulant changes in frozen treated platelet membrane surfaces [4].

Recently, it was also reported that fresh PCs with almost expired shelf lives contained platelet-derived microparticles with 50 to 100 times more potent procoagulant activity than activated platelets and they had a significant impact on the activation and continuation of the coagulation cascade [11,12]. In 2014, Johnson et al. demonstrated increased phosphatidylserine expression on cryopreserved platelet membranes and also showed that these cryopreserved APCs contained phosphatidylserine microparticles that might contribute to the increased hemostatic activity. They also presented the first in vitro phosphatidylserine-dependent coagulation and thrombin generation potentials of cryopreserved APCs by using the TGT [13].

The use of autologous plasma was the most common method for resuspending PCs after thawing until 2006 when Valeri et al. claimed that 0.9% NaCl could be used instead of autologous plasma [14]. The design of this study includes 2 different dilution groups (0.9% NaCl and autologous plasma), aimed to better delineate the dilution method that meets the quality control criteria.

According to the Guide to the Preparation, Use and Quality Assurance of Blood Components: European Directorate for the Quality of Medicines & Healthcare of the Council of Europe (EDQM), frozen APCs have 3 quality control criteria (platelet recovery, residual leukocyte count, and volume) [15]. The platelet recovery rate should be higher than 40%. In our study, the mean platelet recovery rate in the plasma-diluted group (88.1±9.5%) was higher than in the 0.9% NaCl-diluted group (63±10%). However, the platelet recovery rate of both dilution groups met the EDQM quality criteria. Compared to other studies, the results of the 0.9% NaCl-diluted group were lower than in Valeri et al.’s study (74±11%), but those of the plasma-diluted group were better than in Lelkens et al.’s (77±15%) [16,17]. One potential weakness in the current study is the lower number of samples assessed.

The EDQM’s criteria require that the volume of post-thaw APCs be ≥50 mL. In our study, the mean volume of APCs after thawing and dilution was 45±3 mL, which was due to the division of 200 mL of APCs into 2 packs of equal volume prior to freezing. Another quality standard requires the presence of <1x106 residual leukocytes in post-thaw APCs, which was met by all the samples in both dilution groups [15].

The maximum shelf life of PCs stored at 22 °C is 5-7 days. Below this temperature, toxic effects begin to appear in the cells. Moreover, ice crystal formation occurs at low-temperature storage (<0 °C). This formation can puncture the platelet membrane, leading to cell death. In this study, we aimed to show the effect of low temperature on the viability of platelets by flow-cytometric analysis. The mean viability rates of post-thaw APC samples diluted with plasma and 0.9% NaCl were determined as 94.9% and 96.6%, respectively (Table 2). These results revealed that toxic effects of temperature could be prevented using DMSO as a cryoprotective agent.

The TGT is an assay that measures the overall tendency of thrombin formation after initiation of coagulation [18]. The use of autologous plasma for the dilution of frozen thawed platelets could affect the TGT test results. Frozen APCs diluted with autologous plasma (Figure 1A) had correspondingly higher thrombin generation potentials, as in Johnson et al.’s study [13], when compared to both fresh platelets (Figure 1B) and the APC group diluted with 0.9% NaCl (Figure 1C). These findings suggest that cryopreservation increases the platelet hemostatic activities independently from the plasma content. Due to the presence of coagulation factors, the plasma content may also provide an additional hemostatic stimulus when compared to the 0.9% NaCl-diluted group.

Platelet aggregation responses were negative as expected. In Valeri et al.’s study, frozen treated platelets had a significant decrease in aggregation response irrespective of the dilution or resuspension method when compared to fresh platelets (p<0.001) [2]. On the other hand, Hornsey et al. reported that frozen treated platelets demonstrated no aggregation response [19]. However, DMSO-treated platelets were effective on kidney bleeding time in a study that investigated the correlation of in vivo and in vitro functions of fresh and stored human platelets [20].

DMSO-treated frozen platelets have been used successfully since the 1970s [1,17,21]. Khuri et al. compared the clinical effects and hemostatic efficiency of frozen and liquid-preserved platelets in patients undergoing cardiopulmonary bypass in 1999 [3]. They reported that cryopreserved platelet transfusions were superior to liquid-preserved platelets in reducing blood loss and blood transfusion requirements after cardiopulmonary bypass. They concluded that these results were probably related to the improved in vivo hemostatic activity of cryopreserved platelets [3].

In 2001, Özsan et al. studied the cryopreservation of platelets by using a cryopreserving agent and showed that sialic acid was not an alternative compound for cryopreservation [22]. In 2003, Kurt Yüksel et al. presented a case report that demonstrated that the autologous transfusion of cryopreserved platelets could be a reasonable approach in bleeding alloimmunized patients [23].

In 2001, the Netherlands Military Blood Bank implemented the use of frozen platelets in Bosnia and abandoned the walking blood bank concept. Within a 6-month period, 2 thrombocytopenic casualties with exsanguinating hemorrhage were treated with frozen platelets. After those reports, frozen platelets and frozen blood bank facilities became an essential part of military hospital standard equipment deployed by the military of the Netherlands [17]. In 2008, the Australian Defence Force embedded a surgical and intensive care team into the Netherlands-led forward health facility in Afghanistan. Twenty-two units of frozen platelets were used by these teams for 17 casualties undergoing surgery. Except in one patient, there was no clinical evidence of coagulopathy in patients treated with frozen platelets [24]. Between 2006 and 2012, 6246 cryopreserved blood products were transfused in Afghanistan; 2175 of them were erythrocyte concentrations, 3001 were fresh frozen plasma, and 1070 were frozen platelets. No transfusion reactions were reported related to the use of these cryopreserved blood components [25].

The cryopreservation of platelets increases their shelf life from 7 days to 2 years when they are stored at -80 °C with the cryoprotective agent DMSO. Difficulties in the preparation of APCs and the cost of sets could be prevented with the use of cryopreserved blood products. Moreover, the availability of autologous cryopreserved platelets for patients likely to develop refractoriness to platelets or allogenic ABO- and human leukocyte antigen-compatible cryopreserved platelets is crucial for the treatment of these patients. The strategic location of Turkey mandates the urgent collaboration of the Turkish Armed Forces, the Red Crescent, and other governmental medical organizations in establishing both frozen platelet and erythrocyte stocks.

### Ethics

Ethics Committee Approval: Gülhane Military Medical Academy Ethics Committee (Approval number: 06-05-14/37), Informed Consent: It was taken.

## Figures and Tables

**Table 1:**
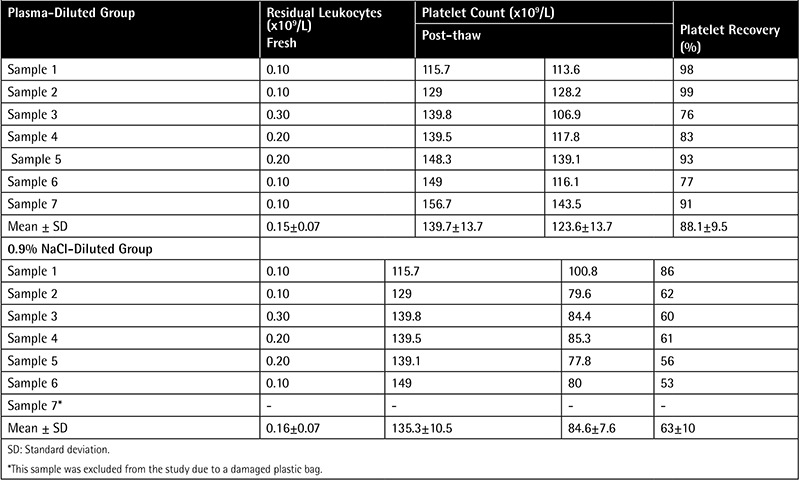
Residual leukocyte and platelet counts, and platelet recovery rates of fresh apheresis platelet concentrates and apheresis platelet concentrates after thawing.

**Table 2:**
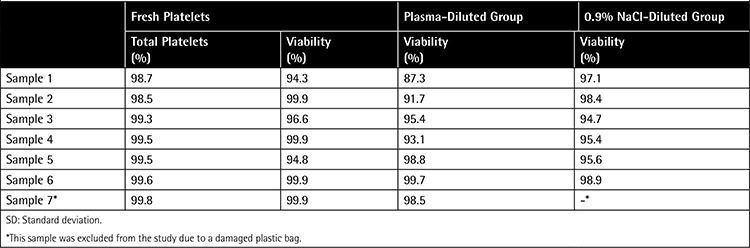
Viability rates of fresh and post-thaw apheresis platelet concentrates.

**Figure 1A:**
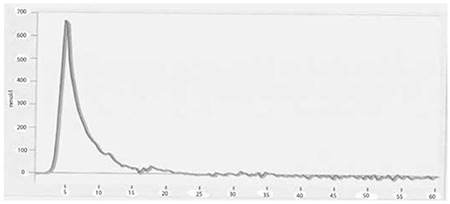
Thrombin generation test results of plasma diluted group (sample). Endogenous thrombin potential and peak height values of apheresis platelet concentrates were 2411 nmol/L per minute and 609 nmol/L, respectively.

**Figure 1B:**
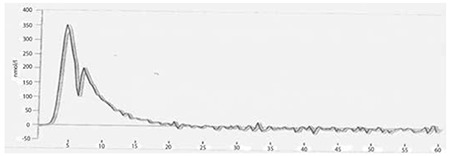
Thrombin generation test results of apheresis platelet concentrates before freezing (sample). Endogenous thrombin potential and peak height values of apheresis platelet concentrates were 1681 nmol/L per minute and 350 nmol/L, respectively.

**Figure 1C:**
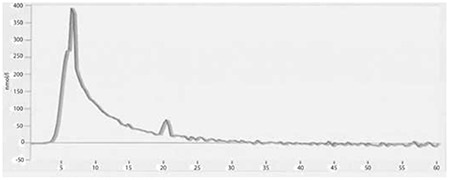
Thrombin generation test results of 0.9% NaCl-diluted group (sample). Endogenous thrombin potential and peak height values of apheresis platelet concentrates were 1913 nmol/L per minute and 338 nmol/L, respectively.
